# The Significance of Lipids to Biofilm Formation in *Candida albicans*: An Emerging Perspective

**DOI:** 10.3390/jof4040140

**Published:** 2018-12-18

**Authors:** Darakshan Alim, Shabnam Sircaik, Sneh Lata Panwar

**Affiliations:** Yeast Molecular Genetics Laboratory, School of Life Sciences, Jawaharlal Nehru University, New Delhi 110067, India; darakshan.alim@gmail.com (D.A.); sircaikshab@gmail.com (S.S.)

**Keywords:** *Candida albicans*, biofilm, lipids, extracellular matrix, drug resistance, antifungal therapy, flippase, mixed-species biofilm, plasma membrane, transcription factors

## Abstract

*Candida albicans*, the dimorphic opportunistic human fungal pathogen, is capable of forming highly drug-resistant biofilms in the human host. Formation of biofilm is a multistep and multiregulatory process involving various adaptive mechanisms. The ability of cells in a biofilm to alter membrane lipid composition is one such adaptation crucial for biofilm development in *C. albicans*. Lipids modulate mixed species biofilm formation in vivo and inherent antifungal resistance associated with these organized communities. Cells in *C. albicans* biofilms display phase-dependent changes in phospholipid classes and in levels of lipid raft formation. Systematic studies with genetically modified strains in which the membrane phospholipid composition can be manipulated are limited in *C. albicans*. In this review, we summarize the knowledge accumulated on the impact that alterations in phospholipids may have on the biofilm forming ability of *C. albicans* in the human host. This review may provide the requisite impetus to analyze lipids from a therapeutic standpoint in managing *C. albicans* biofilms.

## 1. Introduction

A large number of microorganisms are capable of growing both as planktonic as well as in sessile, complex communities surrounded with extracellular matrix called biofilms. The ability to form biofilms has enabled microorganisms to survive and flourish in every niche of earth. Formation of bacterial biofilms was extensively studied and known since 17th century by Antonie Van Leeuwenhoek who discovered bacterial biofilm on his own teeth [1]. Later, discovery of electron microscopy paved way for obtaining a better insight of the detailed structure of biofilm [2]. Biofilms made by bacterial species such as *Pseudomonas aeruginosa* and *Staphylococcus aureus* were extensively studied [3]. Apart from bacteria, fungi such as *Candida*, *Cryptococcus*, *Aspergillus*, *Pneumocystis*, and *Coccidioides* are also known to form biofilm [4]. Amongst the pathogenic *Candida* species, *Candida albicans* is one of the leading causes of hospital-acquired infections [5]. Though, it resides as a harmless commensal in the human host where it colonizes asymptomatically to different niches such as the reproductive tract, gastrointestinal tract, mouth and skin, conditions such as pH or oxygen changes, use of antibiotics or immunosuppressant therapy triggers it to switch to the pathogenic form [6]. The ability of *C. albicans* to form biofilm poses a significant medical challenge in the treatment of candidiasis as these structured communities are recalcitrant to treatment by antifungals. *C. albicans* grows as a biofilm on implanted devices such as catheters, dentures, and prostheses in the human host. Biofilms once formed serve as a source of infectious cells leading to disseminated bloodstream and invasive systemic infections. Over half of the central venous catheters that are placed in individuals are susceptible to biofilm-based *Candida* infections. The treatment of these drug-resistant biofilm based infections is highly expensive as it requires high doses of antifungals and eventually may result in the removal of these devices. Additionally, treatment with high doses of antifungals often leads to other complications in critically ill patients. The increased prevalence of biofilm based infections heralds a need for biofilm-specific drugs and novel strategies for identifying better drug targets. 

In recent years, a considerable number of studies have emphasized the contribution of lipids to fungal pathogenicity and other virulence mechanisms such as drug resistance, biofilm formation, and release of extracellular vesicles [7]. Lipid microdomains composed of sphingolipid-ergosterol molecules have been demonstrated to affect physical properties of cell membrane, thereby contributing to virulence. Additionally, lipids have also been identified as an important constituent of the extracellular matrix (ECM) that protects fungal biofilms. Various studies with bacteria demonstrate lipids as determinants of cell attachment to surfaces and biofilm formation [8]. In the fungal context, studies pertaining to lipid and their contribution to biofilm development are preliminary and limited to date. Nevertheless, an insight into the impact of lipids on fungal biofilm formation will not only increase our understanding of fungal pathogenesis but also may have therapeutic implications. This review begins with a general description of biofilm development and its regulation, followed by summary of studies performed to identify the contribution of lipids in fungal biofilm development.

## 2. Biofilm Architecture, Development, and Regulation in *C. albicans*

**Figure 1:**
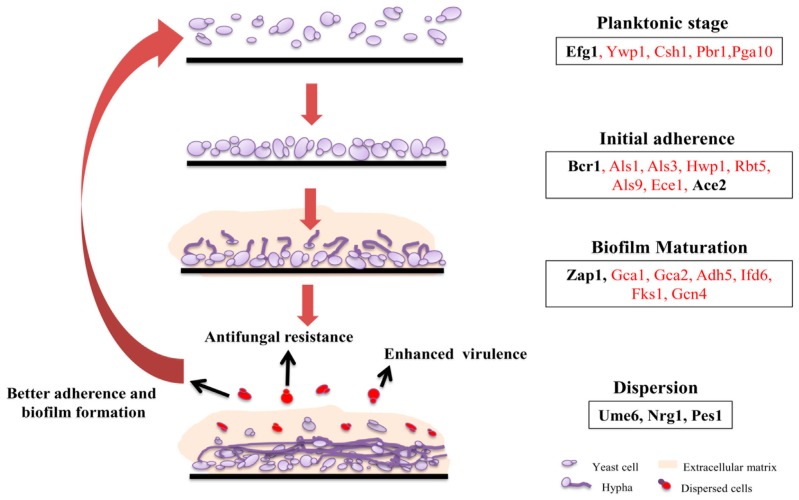
Biofilm development phases in *C. albicans*. *C. albicans* biofilm life cycle comprises of attachment of free *C. albicans* cells to the surface, formation of hyphae, extracellular matrix production, and detachment (dispersion) of cells that can initiate biofilm formation at new sites. For simplicity, few genes including transcription factors (in bold) involved in the indicated stages (as identified in both in vitro and in vivo conditions) are presented in the box. Arrows indicate the properties of the dispersed yeast cells such as enhanced adherence, virulence, and antifungal resistance.

Hawser and Douglas in 1994, for the first time, demonstrated in vitro model system to study biofilm in *Candida* species [9]. The basic idea of the temporal development of biofilm was established by studying in vitro biofilm models using bioprosthetic materials such as polymethylmethacrylate and silicon elastomere [9,10]. As substantiated from in vitro studies, formation of biofilm comprises of four different steps i.e., adhesion, proliferation, maturation, and dispersion [9,10,11,12,13,14] (Figure 1). The first and the foremost step is the adherence of yeast cells to the surface as well as with each other, thus forming a basal layer of biofilm. Followed by adherence, the cells proliferate forming an anchor layer thus providing primary stability to biofilm. Maturation of biofilm takes typically 24 h in an in vitro system. A mature biofilm comprises of mixed type of cells, i.e., round and spherical yeast cells with filamentous hyphae and pseudohyphae intertwined with each other surrounded with dense extracellular matrix. The filamentous, long hyphae are crucial for providing the structural stability to biofilm, holding the yeast cells and extracellular matrix together [6]. Early phase of biofilm lacks ECM, whereas, progression towards a mature biofilm is marked by dense ECM [10]. ECM functions as a cushion preventing the biofilm from any physical perturbations and in providing resistance against various xenobiotics [15]. The final step in biofilm formation is marked by the release and dispersal of yeast cells to new sites, thus making the infection systemic. Dispersion occurs throughout the process of biofilm formation but is seen to be more profound during the intermediate stage of biofilm formation [16]. These dispersed yeast cells differ markedly from planktonic cells, with enhanced virulence and adhesive characteristics, thus making them fit to colonize and establish new biofilms [16].

Formation of biofilm is a complex and multistep process and hence controlled by a wide array of transcription regulators (Figure 1). Transcription factor deletion library was screened for deficiency in biofilm formation to gain an idea about the regulatory network. Of the total 165 mutant strains, 6 mutants were found to be exclusively responsible for biofilm formation and did not show a general growth defect [17]. These six transcription factors (Bcr1, Tec1, Efg1, Ndt80, Rob1, Brg1) form the core biofilm regulators. Although these mutants were unable to develop biofilms, except for Efg1, all other mutants could make normal hyphae in biofilm forming media as well as in suspension cultures [17]. The two currently available in vivo systems to study biofilm formation, rat venous catheter [18] and rat denture models [19] were utilized to assess the effects of these mutants in the in vivo condition, given the fact that biofilm formation in vivo will differ significantly from biofilm formation in vitro due to addition of several factors in the in vivo condition [20]. All the mutants were defective in biofilm formation in rat catheter model, though Brg1 mutant had some kind of morphologically distinct formation of adherent cells and extracellular matrix [17]. However, in rat denture model, Bcr1 mutant could overcome the defects and form biofilms to some extent. This variation in the phenotype observed in both models could be due to the influence of different environmental conditions. In later studies, Fox et al. identified three additional master regulators, namely, Flo8, Gal4, and Rfx2. Deletion mutants of Gal4 and Rfx2 showed enhanced biofilm formation thus acting as negative regulators of biofilm development [21]. To identify the direct downstream effectors of all these nine transcription factors, full genome chromatin immunoprecipitation microarray (CHIP-chip) was performed, which led to some interesting revelations. A total of about 1000 genes are regulated by these biofilm transcription factors and each of the regulator has an influence on the effectors of others as well as on the expression of each other [17,21]. The genetics of each step of biofilm formation and the regulators involved therein is described in detail in the following sections.

### 2.1. Adherence

Adhesion is the initial step in the formation of *C. albicans* biofilms, which typically takes about 1–2 h in an in vitro system. Although current understanding about the mode of adhesion is incomplete, recent studies have indicated an array of conditions critical for this process. These conditions range from nature of the surface to the presence of interacting microorganisms [22,23]. Even host hormones have been found to affect biofilm formation [24]. Microarray studies of cells participating in biofilm formation were compared with un-adhered cells after the adhesion process or cells growing in suspension at log phase or stationary phase to find genes having altered expression. This resulted in the identification of a series of genes with expressions altered in a temporal fashion [21]. A total of 408 genes were identified to be upregulated or downregulated at least two-fold in adhered cells. More than half of these upregulated genes correspond to components involved in DNA synthesis, RNA synthesis, and processing and protein synthesis, suggesting preparation for drastic morphological and physiological shift upon physical adherence [21]. There was also upregulation in two classes of adhesion molecules, one at early point of time while the other at later time point. The early induced class is a group of 10 adhesion molecules, namely, *ALS1*, *ALS2*, *ALS3 ALS4*, *EAP1*, *MSB2*, *PGA6*, *SIM1*, *ORF19.2449*, and *ORF19.5126*), seven of which were unique to only adhered cells suggesting its involvement in attaching to the substrate. The remaining three *(MSB2*, *ALS3*, and *ORF19.2449*) were also upregulated in un-adhered cells indicating their induction was not dependent on contact with a solid surface [21]. Further screening by using deletion strains revealed that five of these early induced genes—*ALS1, ALS2, ALS3, EAP1*, and *MSB2*—are directly associated with biofilm formation [14,17,25,26,27,28]. The other class of adhesion molecules (*HYR1*, FAV2, *IFF4*, *IFF6*, *PGA32*, *PGA55*, *ORF19.3988*, *ORF19.4906*, *ORF19.5813*, and *ORF19.7539.1*) upregulated at later point of biofilm formation are assumed to function in cell-cell adhesion in mature biofilms [21].

The aforementioned genes are only a few of the many components that are involved in the complex mechanism of adherence, some of which have been identified while others require further study. In vitro adherence experiment using silicon as a surface identified more than 30 transcription regulators involved in this process [28]. Among these, the transcription factor Bcr1 is a prominent one as it is required for adhesion to all kinds of surfaces [29]. One important change that happens upon adherence is that there is a global decrease in the expression of metabolic genes, similar to what happens in stationary cells, albeit to a greater extent [21].

### 2.2. Formation of Hyphal Cells

The next crucial step in biofilm formation is the transformation of the yeast cells to hyphal cells, which typically starts after 4–6 h from biofilm initiation. *C. albicans* yeast cells in suspension culture can be induced to form hyphal cells simply by increasing the culture temperature from 30˚C to 37˚C, change in pH, CO_2_, and O_2_ levels [30]. Many transcription factors and other components involved in hyphal formation during biofilm development are also involved in hyphal formation in suspension culture system [12,17]. Some of the master regulators of biofilm formation such as Efg1, Rob1, Ndt80, and Tec1 are also known to regulate hyphae formation in planktonic cells. Bcr1, the master regulator of biofilm is not directly involved in hyphae formation but in adherence of hyphae to each other [29]. Furthermore, kinase deletion planktonic cells defective in hyphal cell formation also were defective in biofilm formation [31]. Hyphae provide a scaffold to yeast cells and extracellular matrix, thus enabling the biofilm to acquire a thicker, resilient structure. Studies also show that clinical strains having high biofilm forming ability has increased expression of hyphae specific genes, confirming the significance of hyphae in biofilm formation [32]. 

### 2.3. Extracellular Matrix Production

The hyphal transition is followed by the production of extracellular matrix (ECM). The dense extracellular matrix encasing mature biofilms are composed primarily of glycoproteins and carbohydrates along with lipids and nucleic acids [15]. Its chemical nature suggests that it is less likely formed by secretion of components alone, but rather by lysis of some of its own cells along with other components [15]. One of the key factors contributing towards the increased antifungal resistance to biofilm is the presence of extracellular matrix [33]. β1-3 glucan is known to provide resistance against fluconazole by sequestering the drug [34]. Rlm1 and Zap1 are the two transcriptional regulators that regulate the matrix production [35,36]. Zap1 negatively regulates the production of β1-3 glucan. The target genes of Zap1 includes *GCA1* and *GCA2*, the known glucoamylase [35]. Rlm1 positively regulates the production of β1-3 glucan through the modulation of *FKS1*, the glucan synthase [34]. Interestingly, the extracellular DNA present in the ECM is shown to contribute to the overall stability of biofilm as treating ECM with DNase led to decrease in biofilm biomass at later time point [37].

### 2.4. Dispersion

Dispersion of yeast form of *C. albicans* occurs during and after the formation of biofilms [16]. It is a way used by the pathogen to spread through blood stream and forming biofilms at new sites, thus making the infection systemic. Several components such as transcriptional regulators, cell wall proteins, chaperons, etc. are crucial for this step [38,39,40]. Ume6, Pes1, and Nrg1 are the known regulators that are involved in the dispersion of cells from biofilm. Overexpression of the known regulator of hyphal extension, *UME6* [41] decreases dispersion. On the contrary overexpression of *NRG1*, a negative regulator of filamentation [42] and *PES1*, regulator of the transition of hyphae to yeast form [43], increases the dispersal of cells from biofilm [16].

## 3. Structural and Functional Contribution of Lipids to Biofilm Formation

Considerable evidence in the recent years has shown that lipids present on the plasma membrane and ECM may be essential for the architectural stability of the biofilm [7,44]. While the genetic control of biofilm development is well-documented [21], molecular studies focusing on the contribution of lipids to ECM biogenesis and traits associated with biofilms are restricted in *C. albicans*. It has been demonstrated that lipids are critical in modulating microbial biofilms as cells in a biofilm differ from those in the planktonic growth mode in the distribution of phospholipids and their molecular species. Variation in the sterol and sphingolipid profile between the planktonic and biofilm cells affects not only the ability of *C. albicans* to form biofilms but also influences antifungal resistance associated with this structured community [45]. Furthermore, membrane lipid composition is considered an important determinant of cell size and cell shape; essential morphological parameters that ensure surface adhesion and biofilm formation in bacterial cells [8]. Concordantly, it is conceivable that dynamic changes in lipid profiles may also have detrimental effects on cellular shape and cell physiology in *C. albicans*. As a result, phospholipid-altered *C. albicans* strains may exhibit impaired adhesion, leading to a defect in biofilm formation, an area that can be harnessed for future antifungal therapy. In the following sections, a review of the literature on the contribution of lipids to the structure and function of biofilms is discussed.

### 3.1. Lipids are Constituents of Fungal Membrane 

**Figure 2:**
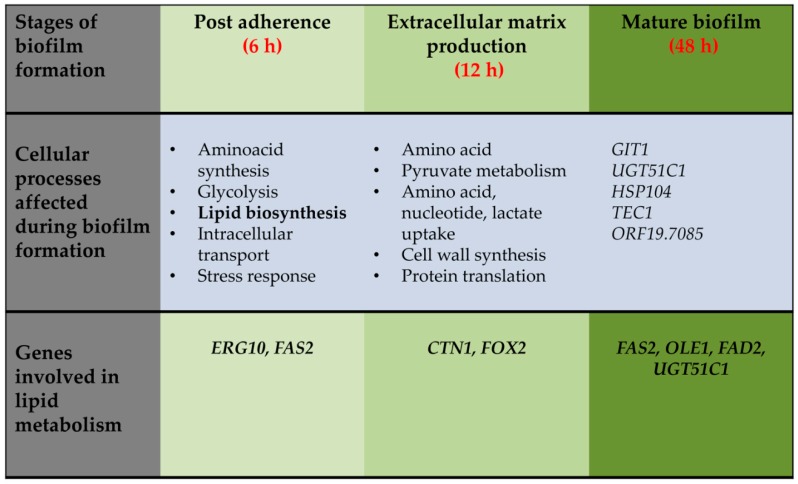
Cellular processes upregulated during biofilm formation at different time interval. Time course for upregulation of the listed cellular processed is shown as inferred from the transcriptome analysis reported by Yeater et al., 2007 [50]. Differentially regulated genes associated with lipid homeostasis during different phases of biofilm development are also shown. For simplicity, categories of genes upregulated at 6 h vs. 12 h are summarized under the 6 h heading whereas, data from the 12 h vs. 6 h and 12 h vs. 48 h comparisons are placed under 12 h heading. Individual genes upregulated at 48 h vs. 12 h are listed under 48 h section.

Considering the role of lipids in providing structural integrity to the plasma membrane and in modulating fungal growth and pathogenesis [46,47,48], studies have been performed in *C. albicans* to understand the importance of lipids during the early and mature phases of biofilm. These studies involved (i) comparison of transcriptional profiles of *C. albicans* biofilm (formed on denture and catheter substrates) and planktonic cultures as a function of time and (ii) two-dimensional difference-in-gel electrophoresis (DIGE)-based proteomics on cell walls and ECM from biofilms and planktonic cells [49,50,51]. The microarray analysis examined three biofilm developmental phases—early (6 h), intermediate (12 h), and mature (48 h)—and compared it with planktonic cells grown to the same time points. Glycolysis/gluconeogenesis is one of the most common pathway that is differentially regulated in the early as well as the mature phases of biofilm development. Additionally, pathways such as the pentose phosphate pathway, TCA cycle, amino acid metabolism, sterol, fatty acid, and lipid metabolism are also upregulated during biofilm formation [49] (Figure 2). All these processes occur at the early time point and are necessary to build up levels of biomolecules that are required for the biomass increase during the intermediate phase of biofilm development. Initiation of new metabolic activity ceases during the mature phase of biofilm as indicated by the differential regulation of few genes in that phase (Figure 2) [49,50]. 

The differential expression of genes involved in lipid biosynthesis pointed to a role of lipids in biofilm formation. Subsequently, lipidome analysis of biofilm and planktonic cells confirmed the impression that lipids may exert an influence on the biology of *C. albicans* biofilms [52]. Lipid profiling indicated towards a growth-phase-dependent difference in the levels of the phospholipid, sphingolipid and sterol species in biofilms. Cells in the biofilm mode of growth are enriched in all polar lipids especially phosphatidylinositol (PI). The levels of phosphatidylcholine (PC), phosphatidylethanolamine (PE), phosphatidylinositol (PI), phosphatidylserine (PS), and phosphatidic acid (PA) are higher in the early and mature phases of biofilm formation, compared to the planktonic cells (Table 1). Additionally, the cells in a biofilm display a larger increase in the PC:PE ratio (2-fold) as opposed to a small increase (0.2-fold) in the planktonic cells, in both early and late phases of biofilm development. Furthermore, all molecular species for PC, PI, PE, PS, PA, and PG are higher in biofilms than in planktonic cells as deduced from ESI-MS/MS analysis. The unsaturation indices of various species of lipids also vary in the biofilm as reflected in the high unsaturation index observed for PC and PS in early-phase biofilms versus low index of PC, PE, and PS in mature phase of biofilms. In contrast, the planktonic cells do not display a significant change in the unsaturation indices of various phospholipid classes. Biofilm cells also exhibit high levels of sphingolipid species [52], in accord with the increased levels of PI, which is involved in biosynthesis of sphingolipids, in the early-phase of biofilms than in early-phase planktonic cells (Table 1).

Mukherjee and group performed gas–liquid chromatography (GLC) to obtain an insight into the levels of sterols between planktonic and biofilm cells [45] as (i) sterols are also crucial components of the plasma membrane and (ii) alterations in sterol levels have been known to be associated with antifungal resistance in planktonic cells [53]. Their results show that while the levels of the intermediates of the ergosterol biosynthesis pathway remain unaffected in planktonic cells, their levels vary between the developmental phases of a biofilm. Both biofilm and planktonic *C. albicans* show similar ergosterol levels at 6 h followed by 41% and 50% reduction in ergosterol levels in the intermediate and mature phase of biofilms, respectively, compared to the 6 h time point. An 18% reduction in ergosterol levels in both the intermediate and mature phase of planktonic cells indicates that the decrease in ergosterol as the biofilm matures is larger, compared to the planktonic cells. Collectively, these studies suggest that cells in *C. albicans* biofilms exhibit changes in levels of phospholipids, sphingolipid, and sterols presumably to adapt and adjust to varying requirements during the development of a biofilm. The overall effect of changes in sterol and sphingolipid levels have been shown to prevent biofilm formation in *C. albicans*, suggesting the essentiality of membrane lipids in this cellular process [45,52,54]. 

### 3.2. Lipids Are Constituents of Extracellular Matrix

Lipids have also been found to be associated with the *C. albicans* biofilm matrix. Given the importance of ECM in contributing to an array of functions [10,44,55,56], studies pertaining to its structure, composition, and function have gained recent interest. Douglas et al. in the year 2000 described a basic protocol for matrix extraction and analyzed its constituents [56]. Since then several approaches have been employed to isolate and study a pure biofilm matrix devoid of any intracellular component. The recent protocol for matrix extraction established by Andes group deploys a large-scale extraction method with subsequent filtration, dialysis, and lyophilization procedures yielding the most purified form of matrix suitable for its biochemical and functional analyses [57].

**Figure 3:**
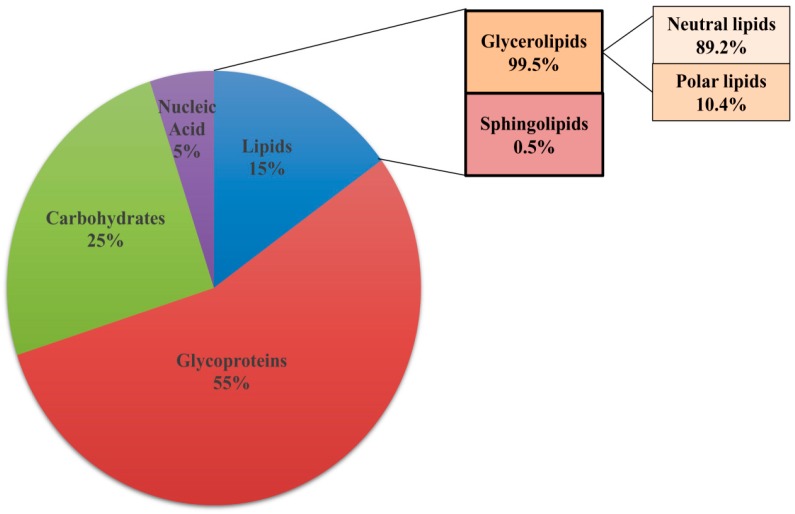
Composition of extracellular matrix (ECM) of biofilm. ECM of biofilm comprises of glycoproteins (55%), carbohydrates (25%), lipids (15%), and nucleic acid (5%). Glycerolipids (99.5%) and sphingolipids (0.5%) are the different classes of lipids present in the ECM. Glycerolipids contain a mixture of neutral lipids and polar lipids.

Biochemical analysis shows that lipids constitute 15% of the total dry weight of the ECM along with 55% glycoproteins, 25% carbohydrates and 5% nucleic acids (Figure 3). Gas chromatography revealed the presence of eight different classes of lipids in the matrix, with a prevalence of glycerolipids (99.5%) while sphingolipids constitute only 0.5% of the total matrix lipid [15]. Glycerolipids comprise of neutral lipids (89.2%) with small amount of polar lipids (10.4%). The fatty acid side chains on these lipids were classified into 13 different classes. Amongst the fatty acids present in the neutral lipids, oleic acid and linoleic acid were in maximum proportion with small amounts of palmitic acid, stearic acid, palmitoleic acid, and myristic acid. The polar lipids present in the biofilm matrix consist largely of PE (8.9%) with little amounts of PC, PI, and sphingomyelin. Mass spectroscopy also revealed the presence of ergosterol and prostaglandin E2 in small amounts [15]. The exact role of the various lipid species present in the ECM in the context of matirx biogenesis and biofilm development has not been investigated. Exploring the relevance of all the components of ECM will pave a way for a better understanding of the contribution of lipids to biofilm and its prevention. 

### 3.3. Lipids Facilitate Formation of Lipid Rafts

The above sections describe the structural role of lipids in *C. albicans* biofilm. Another mechanism by which lipids in the plasma membrane of *C. albicans* contribute to biofilm formation is by facilitating formation of microdomains referred to as lipid rafts. These microdomains enriched in sphingolipids and ergosterol are considered pivotal for maintaining the integrity of the plasma membrane and in segregating proteins localized to the plasma membrane [7]. The phase-specific changes in lipid levels during biofilm development impacts the biofilm-forming ability of this pathogenic fungus by perturbing raft formation, suggesting that lipid rafts are essential for biofilm development [52]. *C. albicans* biofilms contain large number of lipid rafts, in concurrence with the high levels of sphingolipids in the biofilm cells, compared to the planktonic cells [52]. Treatment with drugs that inhibit sphingolipid biosynthesis pathway disrupts raft formation followed by reduction in the biofilm-forming ability of *C. albicans*, forging a link between lipid rafts and biofilm formation. Similarly, simvastatin (compound inhibiting ergosterol biosynthesis)-treated *C. albicans* fails to form biofilms, thus tying ergosterol levels to biofilm development. Therefore, inhibiting the sphingolipid or ergosterol biosynthesis pathways pharmacologically or genetically may prove beneficial for illuminating the effect of lipid rafts on biofilm formation in *C. albicans* [52].

### 3.4. Lipid Signaling Modulates Biofilm Formation

Lipids are also known to promote cell-cell signaling, during *C. albicans* biofilm formation [58]. The importance of lipid signaling in regulating fungal pathogenicity has already been established [59]. Farnesol, a quorum sensing lipid molecule, is known to inhibit filamentation, a virulence trait, essential for pathogenesis. Farnesol exerts its effect on biofilm formation by inhibiting the bud-to-hyphae transition; a feature that is fundamental for biofilm development [58,60,61]. The antibiofilm activity of farnesol is observed exclusively during initial time of adherence, with no effect on biofilm formation 1–2 h after adherence and after the commencement of hyphae formation. Furthermore, farnesol that is produced by mature biofilms helps in preventing overpopulation by shifting the morphology to the yeast form, which might disperse to a new site for initiation of new biofilm [61].

Farnesol also acts as a modulator of drug efflux pumps Cdr1 and Cdr2, potent contributors of multidrug resistance (MDR), by competitively inhibiting the efflux of rhodamine 6G. It functions synergistically with antifungals such as fluconazole, miconazole, ketoconazole, and amphotericin in in vitro biofilm models and can be used in combinational therapy to treat drug-resistant biofilms [62]. Thus, albeit the need to further investigate the full potential of farnesol in antifungal therapeutics, it may still be exploited as a prospective target for developing anti-infective strategies in lieu of its role in quorum-sensing in fungal biofilms. 

Prostaglandins are another class of signaling lipids involved in inflammatory and immune responses [63]. Prostaglandins are lipid derivatives of arachidonic acid produced via two cyclooxygenase (COX) enzyme, *COX1* and *COX2*, in mammalian cells. *C. albicans* synthesizes prostaglandin de novo as well as from external arachidonic acid [64]. Increased production of the prostaglandin PGE_2_ in *C. albicans* biofilms hinted towards a regulatory role of this lipid in biofilm formation [65]. Recently, the role of prostaglandin in promoting mixed biofilm with *Staphylococcus aureus* as described later in this review, has also been established [66]. Though cyclooxygenase is not yet identified in *C. albicans*, treatment with cycolooxygenase inhibitors such as aspirin, etodolac, and diclofenac significantly decreases the synthesis of prostaglandin and biofilm formation confirming a regulatory role of COX-dependent prostaglandins in biofilm formation [65,67]. Coupled together, the potential of these signaling lipids in modulating biofilm formation can be harnessed for development of antifungal therapies.

## 4. Lipids Influence Clinically-Relevant Traits Associated with Biofilms

Antifungal resistance associated with *C. albicans* biofilm is one of the most clinically-relevant challenge that has to be considered for treating candidiasis. In addition, *C. albicans* colonizes with multiple species of prokaryotes in vivo to form polymicrobial biofilms on medical surfaces [68,69,70]. As the polymicrobial biofilms consist of multiple species of both prokaryotes and eukaryotes, multiple drug combinations are required to eradicate both fungal and bacterial species [68]. Polymicrobial interactions therefore are likely to dictate the final outcome of treatment with antifungals. Thus, directing therapies to combat the multiplicity of the species and antifungal resistance of a fungal biofilm is of utmost clinical importance in future. In the following section, we review the importance of lipids in imparting antifungal resistance and in promoting mixed biofilms, which may prove beneficial in developing strategies to prevent biofilm formation.

### 4.1. Role of Lipids in Antifungal Drug Resistance

It is reported that biofilms display innate resistance against triazoles displaying up to 1000-fold increased resistance than the planktonic cells, rendering azoles ineffective in treating biofilms [71]. On the other hand, the emergence of drug-resistant planktonic cells is an outcome of long-term treatment of *Candida* infection by triazoles [72,73]. Upregulation of drug efflux pumps (Cdr1, Cdr2, and Mdr1) and mutations in the *ERG11* gene are the most prominent mechanisms that contribute to the development of drug resistance in planktonic cells [74,75,76,77,78,79,80]. Triazoles such as fluconazole are the most common antifungals used to treat *Candida* infections that target *ERG11* gene, encoding lanosterol demethylase. It disrupts the ergosterol biosynthesis pathway leading to the accumulation of 14α-methyl sterol precursor [81,82] in both planktonic and biofilm cells. Considering the involvement of drug efflux pumps in acquiring drug resistance in the planktonic cells, the role of these efflux pumps in imparting antifungal resistance to biofilm has also been investigated. The antifungal susceptibility assay of early biofilms (6 h) made up of the single, double, and triple mutants of *CDR1*, *CDR2*, and *MDR1* genes showed significantly increased susceptibility to fluconazole as compared to the wild type parent strain, with the triple mutant exhibiting the minimum MIC value (16 µg/mL). On the contrary, later phase of biofilm showed resistance to fluconazole similar to the wild type strain (MIC ≥ 256 µg/mL). Thus, this phase-specific study reveals the contribution of these efflux pumps towards azole-resistant phenotype only in the early phase (6 h) of biofilm formation while ruling out their role in the intermediate (12 h) and mature (24 h) phases of biofilm [45]. 

Drug-resistant planktonic cells also exhibit altered sterol composition correlating sterol levels to triazole resistance [53,83]. Altered sterol levels may impact the entry of the antifungal by influencing membrane permeability, resulting in triazole resistance. This prompted researchers to also investigate this perspective in contributing to drug resistance in biofilms. Transcriptional studies of in vitro biofilm cells demonstrate altered expression of sterol biosynthesis genes compared to the planktonic cells [84]. Additionally, in vivo rat venous catheter model also revealed the increased expression of *ERG25*, encoding a putative C4 methyl sterol oxidase. The proposed role of this enzyme is the C4-demethylation of the ergosterol biosynthesis intermediates, converting lanosterol to non-ergosterol intermediates; thus limiting ergosterol synthesis, which limits the potency of triazoles in treating biofilm-based infections [85]. Studies performed by Mukherjee et al. show that resistance to ergosterol targeting antifungals was attributed to the a significantly reduced sterol levels in the intermediate and mature phase of biofilm, validating the link between sterol composition and triazole resistance in different phases of biofilms [45]. Consequently, these studies suggest that altered sterol levels contribute to the biofilm-associated resistance in *C. albicans*. This area warrants further exploration as an impact of these changes on membrane permeability and its effect on triazole entry in biofilms is lacking.

### 4.2. Role of Lipids in Mixed-Species Biofilm Formation

In addition to the decreased susceptibility of biofilms to antifungals, another challenge that impedes treatment of *Candida* infections is occurrence of polymicrobial biofilms in in vivo conditions rather than monomicrobial. Multiple species interacting synergistically and antagonistically with each other lead to the formation of polymicrobial biofilms [86]. It has also been reported that approximately 27–56% of hospital acquired bloodstream infections of *C. albicans* are polymicrobial [87,88]. *C. albicans* is commonly found associated with bacterial species such as *S. aureus* in blood stream infections [89] and with *Streptococcus gordonii* and *Streptococcus mutans* in dental caries or dental stomatitis [90,91]. *S. aureus* is the third most common organism found in association with candidemia with increased mortality rate in animal model [92]. Prostaglandins secreted by *C. albicans*, serve as signaling molecules to promote the growth of *S. aureus* biofilm as examined in an in vitro biofilm infection model system for *S. aureus* and *C. albicans* [66]. Presence of *C. albicans* enhances the growth rate of *S. aureus* by providing a scaffold to the bacteria [93]. In order to explore the role of prostaglandins in promoting *S. aureus* growth, supernatants of cell free *C. albicans* strains SC5314 and 31883 when incubated with *S. aureus* biofilm greatly enhanced the biofilm growth of *S. aureus* while supernatants from prostaglandin mutants (*ura3*∆/∆*fet31*∆/∆) showed no stimulatory effect on *S. aureus* biofilm [66]. Moreover, treatment with non-specific cyclooxygenase inhibitor indomethacin significantly repressed *S. aureus* growth. These observations establish a potential role of prostaglandin in biofilm development as well as in promoting mixed biofilms with *S. aureus*.

Apart from polymicrobial bloodstream infections, occurrence of these mixed-species biofilm is most prevalent in oral cavities which is a site for different microorganisms to reside. *Candida* associated denture stomatitis, an inflammatory mucosal condition affecting 50–70% of denture wearers, is often found co-associated with *Streptococcus mutans* [94,95]. It has been shown that the presence of *S. mutans* facilitates adhesion of *C. albicans* to the surface [96,97]. Therefore, compounds that can affect these mixed-species biofilms are much more needed. In this context, it is worth mentioning the role of farnesol which is known to have antimicrobial as well as antibiofilm activity [61,98]. Recent studies have demonstrated dose dependent role of farnesol in affecting mixed-species biofilm formation. Higher concentration of this compound shows inhibitory effect on *S. mutans* biofilm while lower doses showed stimulatory effect [99]. Treatment with high concentration of farnesol exhibited an inhibitory effect on this dual biofilm of *C. albicans* and *S. mutans* under in vitro conditions [100]. Thus, extending further focus on these signaling molecules will further help in treating these resilient polymicrobial biofilms.

## 5. Targeting Lipid Biosynthesis Impedes Biofilm Formation

The aforementioned studies provide an unprecedented view of the structural, signaling and metabolic pathways in which lipids participate during biofilm development in *C. albicans*. Although lipids are shown to function as regulators of fungal pathogenicity [101], the precise role of lipid-dependent processes in regulating *C. albicans* biofilm formation remains underexplored. Genetic manipulation and pharmacological modulation of lipid biosynthesis pathways are two strategies that have been exploited to tie lipid alterations to biofilm formation in this fungus. Manipulation of membrane lipid composition by systematically targeting gene coding for lipid biosynthesis enzymes facilitates modulation of endogenous lipid levels. Using this approach *IPT1*, a gene coding for an enzyme that catalyzes the terminal step of sphingolipid biosynthesis was deleted in *C. albicans* [52,102]. The *ipt1*Δ/Δ cells display fewer lipid rafts on the hyphal membrane and reduced biofilm formation [52]. Another study demonstrates a role for fatty acid elongases coding genes, *FEN1* (*ELO2*) and *SUR4* (*ELO3*) that are involved in sphingolipid biosynthesis, in biofilm formation [103]. These genes along with *ELO1* synthesize C14-C16 long chain fatty acids, up to C24 very long chain fatty acids (VLCFA) and C24 or C26 VLCFA. Deletions in *FEN1* and *SUR4* results in the production of short chain fatty acids and leads to reduced sphingolipid levels. SEM analysis of poly-L-lysine coated cover slips inoculated with *fen1*Δ/Δ and *sur4*Δ/Δ mutants show defect in biofilm formation [103]. Furthermore, the loss of de novo phosphatidylethanolamine (PE) and phosphatidylserine (PS) synthesis and its impact on the phospholipidome of *C. albicans* has also been demonstrated [104]. Despite the observation that mutations in genes coding for PS synthase (*CHO1*) and PS decarboxylases (*PSD1* and *PSD2*), enzymes involved in PS and PE synthesis, compromise the virulence of *C. albicans* [105], the mutants have not been tested for their ability to form biofilms. Similarly, the effect of manipulating the ergosterol biosynthesis pathway on biofilm formation has not been examined, undermining the contribution of lipids on biofilm formation in *C. albicans*.

Pharmacological modulation of sphingolipid biosynthesis pathway in *C. albicans* has been achieved by using compounds such as myriocin and aureobasidin A. Interestingly, treatment of wild type *C. albicans* strain with these compounds results in blocking biofilm formation [106]. Furthermore, fluconazole which only moderately affects growth of cells in liquid culture by inhibiting ergosterol biosynthesis has a more pronounced effect in preventing biofilm formation by a variety of both fluconazole-susceptible and -resistant *C. albicans* strains [107]. Fluconazole-treated *C. albicans* cells are able to adhere but cannot form the three-dimensional structure of the biofilms, pointing to a biofilm-specific inhibitory role of fluconazole [107]. 

Combining the existing antifungals with other drugs can also be used as a therapeutic approach to treat biofilm-based infections. Drug combination therapy, considered as the best option to treat invasive infections, not only enhances the efficacy of the drugs but also impedes development of drug resistance [85]. The non-steroidal anti-inflammatory (NSAIDs) drugs—such as aspirin, diclofenac, and ibuprofen—display anti-biofilm activity by blocking prostaglandin synthesis that promotes formation of polymicrobial biofilms [106]. A combination of diclofenac and fluconazole is effective in preventing *C. albicans* biofilm formation on catheters in vivo [108]. Similarly, phytocompounds such as eugenol and cinnamaldehyde sensitize biofilms to treatment with fluconazole by converting this fungistatic azole to a fungicidal drug [109]. Using pharmacological modulators of lipid biosynthesis in combination with other compounds will add-on to the therapeutic values of the lipid-specific antifungals in the future. Coupled together, these studies forge a link between lipid biosynthesis pathways and biofilm formation in *C. albicans*.

Aside from using methods to directly target lipid biosynthesis pathways, indirect methods that may cause alterations in phospholipid composition at the plasma membrane will also help in providing evidence that will link alterations in lipids to biofilm development. Phospholipid-altered bacterial strains exhibit changes in cell morphology, in turn leading to a defect in surface adhesion and biofilm formation [8]. Concordantly, changes in lipid composition mediated by lipid translocases is also shown to alter erythrocytes shape, indicating the importance of translocases in maintaining cell shape [110,111]. P4-type ATPases are one such class of translocases that function as flippases; proteins that direct the inward transbilayer movement of phospholipids across the plasma membrane. Floppases, on the contrary regulate the outward-directed transbilayer movement of phospholipids across the plasma membrane. The concerted action of flippases and floppases leads to the asymmetric distribution of phospholipids on the plasma membrane, generating plasma membrane asymmetry in eukaryotic cells [112,113,114]. Flippase activity of the P4-type ATPases can induce changes in the membrane curvature leading to defects in the cell morphology of the erythrocytes [115]. In line with this, elevated flipping of PC by the P4 ATPase ATP10A in HeLa cells results in altered cell shape and size, delayed adhesion and spreading onto the extracellular matrix, conceivably by enhancing the inward bending of plasma membrane [116]. Enhancement of PC flipping induces an imbalance in the level of lipids between the two leaflets of the membrane bilayer. Consequentially, the plasma membrane tends to bend inwards, perturbing the membrane curvature leading to adhesion defects in HeLa cells expressing ATP10A [117]. Therefore, studies focusing on targeting lipid translocases that function as flippases will also help in understanding the influence of phospholipid alteration at the plasma membrane on cell morphology, surface adhesion, and biofilm development in *C. albicans*.

Such data wherein a connection between phospholipid flippase activity and cell adhesion has been established is not available in *C. albicans*. In this context, it is pertinent to mention the recent identification of Rta3, a 7-transmembrane receptor protein and its roles in regulating the activity of an unidentified PC-specific flippase, adhesion, and biofilm formation in *C. albicans* [118]. Absence of Rta3 causes an increase in the flip movement of PC plausibly by regulating an unidentified PC-specific flippase; thus perturbing the asymmetric distribution of PC across the plasma membrane [118]. *rta3*Δ/Δ cells also exhibit a defect in adhesion that in turn leads to a biofilm defect in *C. albicans*. It is possible that enhanced PC flipping and perturbation in membrane curvature may be the basis for the observed adhesion defect in *rta3*Δ/Δ cells, though experimental evidence to prove this needs to be investigated. Considering that Rta3 and its orthologs are exclusive to fungal kingdom, targeting these genes may have therapeutic implication in the future. Identification of proteins that function as flippases and elucidating the regulatory mechanisms of these flippases may offer novel strategies to address the impact of lipids to biofilm formation in this pathogenic fungus.

## 6. Conclusions and Future Directions

The importance of lipids in maintaining plasma membrane integrity and cell survival has been demonstrated in prokaryotic and eukaryotic cells. However, studies pertaining to analyze the impact of systematic alterations in lipid levels on cell shape and its influence on biofilm formation in *C. albicans* remain limited. Presently, a comprehensive understanding of lipid-dependent cellular processes that would impact biofilm formation is essential. The significance of membrane lipids in maintaining cell morphology is one such cellular process and future area of interest. Changes in cell shape may lead to defects in surface adhesion, thus negatively influencing the biofilm forming ability of *C. albicans*. Further studies on the influence of phospholipid composition on the structure and function of the cell wall/membrane or the ECM and its effect on cell shape warrant more investigations. Identification of lipid translocases of the P4-type and their regulators, their systematic deletion in *C. albicans* may augment our knowledge in this area. Subsequently, these mutant strains can be analyzed for alterations in membrane lipid composition and its effect on cell shape and thus biofilm formation. Deciphering the role of phospholipids in detecting and integrating environmental signals to regulate biofilm formation will prove beneficial for developing antifungal therapies. Considering the lack of biofilm-specific antifungals, identifying novel lipid-dependent cellular pathways crucial for biofilm formation and targeting them may have significant clinical impact in future.

## Figures and Tables

**Table 1:** Distribution of lipids in *C. albicans* biofilms and planktonic cells as gathered from lipidomic analysis by Lattif et al. 2011 [52]. Asterisk (*) represents average lipid levels in biofilms and planktonic cells grown to early phase (6 h) or mature phase (48 h). The number of asterisks indicates the extent of alteration in the levels of the corresponding phospholipid in the given phase. The mean lipid levels were found to be increased in both early and late biofilm, compared to planktonic cells. nd: not detectable.

Lipid Molecular Species		Planktonic Stage		Biofilm Stage	
		Early Phase	Late Phase	Early Phase	Late Phase
Phospholipids	Phosphatidylcholine	*	*	**	**
	Phosphatidylethanolamine	*	*	**	**
	Phosphatidylinositol	*	*	***	***
	Phosphatidylserine	*	*	**	**
	Phosphatidic acid	*	*	**	**
	Phosphatidylglycerol	*	*	**	**
Sphingolipids	Inositolphosphorylceramide	*	*	***	***
	Mannosylinositolphosphorylceramide	*	*	***	***
	Mannosyldiinositolphosphorylceramide	*	***	***	nd

## References

[1] Percival S.L., Malic S., Cruz H., Williams D.W. (2011). Introduction to biofilms. Biofilms and Veterinary Medicine.

[2] Harty D.W., Handley P.S. (1989). Expression of the surface properties of the fibrillar *Streptococcus salivarius* HB and its adhesion deficient mutants grown in continuous culture under glucose limitation. Microbiology.

[3] Allewell N.M. (2016). Introduction to biofilms thematic minireview series. J. Biol. Chem..

[4] Fanning S., Mitchell A.P. (2012). Fungal biofilms. PLoS Pathog..

[5] Kojic E.M., Darouiche R.O. (2004). *Candida* Infections of Medical Devices. Clin. Microbiol. Rev..

[6] Lohse M.B., Gulati M., Johnson A.D., Nobile C.J. (2018). Development and regulation of single-and multi-species *Candida albicans* biofilms. Nat. Rev. Microbiol..

[7] Rella A., Farnoud A.M., Del Poeta M. (2016). Plasma membrane lipids and their role in fungal virulence. Prog. Lipid Res..

[8] Rowlett V.W., Mallampalli V.K., Karlstaedt A., Dowhan W., Taegtmeyer H., Margolin W., Vitrac H. (2017). The impact of membrane phospholipid alterations in *Escherichia coli* on cellular function and bacterial stress adaptation. J. Bacteriol..

[9] Hawser S.P., Douglas L.J. (1994). Biofilm formation by *Candida* species on the surface of catheter materials in vitro. Infect. Immun..

[10] Chandra J., Kuhn D.M., Mukherjee P.K., Hoyer L.L., McCormick T., Ghannoum M.A. (2001). Biofilm formation by the fungal pathogen *Candida albicans*: Development, architecture, and drug resistance. J. Bacteriol..

[11] Baillie G.S., Douglas L.J. (1999). Role of dimorphism in the development of *Candida albicans* biofilms. J. Med. Microbiol..

[12] Ramage G., Vandewalle K., Wickes B.L., López-Ribot J.L. (2001). Characteristics of biofilm formation by *Candida albicans*. Rev. Iberoam. Micol..

[13] Douglas L.J. (2003). *Candida* biofilms and their role in infection. Trends Microbiol..

[14] Nobile C.J., Mitchell A.P. (2006). Genetics and genomics of *Candida albicans* biofilm formation. Cell. Microbiol..

[15] Zarnowski R., Westler W.M., Lacmbouh G.A., Marita J.M., Bothe J.R., Bernhardt J., Sahraoui A.L.-H., Fontaine J., Sanchez H., Hatfield R.D. (2014). Novel entries in a fungal biofilm matrix encyclopedia. MBio.

[16] Uppuluri P., Chaturvedi A.K., Srinivasan A., Banerjee M., Ramasubramaniam A.K., Köhler J.R., Kadosh D., Lopez-Ribot J.L. (2010). Dispersion as an important step in the *Candida albicans* biofilm developmental cycle. PLoS Pathog..

[17] Nobile C.J., Fox E.P., Nett J.E., Sorrells T.R., Mitrovich Q.M., Hernday A.D., Tuch B.B., Andes D.R., Johnson A.D. (2012). A recently evolved transcriptional network controls biofilm development in *Candida albicans*. Cell.

[18] Andes D., Nett J., Oschel P., Albrecht R., Marchillo K., Pitula A. (2004). Development and characterization of an in vivo central venous catheter *Candida albicans* biofilm model. Infect. Immun..

[19] Nett J.E., Marchillo K., Spiegel C.A., Andes D.R. (2010). Development and Validation of an In Vivo *Candida albicans* Biofilm Denture Model. Infect. Immun..

[20] Nett J., Andes D. (2006). *Candida albicans* biofilm development, modeling a host–pathogen interaction. Curr. Opin. Microbiol..

[21] Fox E.P., Bui C.K., Nett J.E., Hartooni N., Mui M.C., Andes D.R., Nobile C.J., Johnson A.D. (2015). An expanded regulatory network temporally controls *C andida albicans* biofilm formation: Expanded biofilm regulatory network. Mol. Microbiol..

[22] Frade J.P., Arthington-Skaggs B.A. (2011). Effect of serum and surface characteristics on *Candida albicans* biofilm formation. Mycoses.

[23] Parsek M.R., Greenberg E.P. (2005). Sociomicrobiology: The connections between quorum sensing and biofilms. Trends Microbiol..

[24] Alves C.T., Silva S., Pereira L., Williams D.W., Azeredo J., Henriques M. (2014). Effect of progesterone on *Candida albicans* vaginal pathogenicity. Int. J. Med. Microbiol..

[25] Zhao X., Oh S.-H., Yeater K.M., Hoyer L.L. (2005). Analysis of the *Candida albicans* Als2p and Als4p adhesins suggests the potential for compensatory function within the Als family. Microbiology.

[26] Li F., Svarovsky M.J., Karlsson A.J., Wagner J.P., Marchillo K., Oshel P., Andes D., Palecek S.P. (2007). Eap1p, an adhesin that mediates *Candida albicans* biofilm formation in vitro and in vivo. Eukaryot. Cell.

[27] Swidergall M., Filler S.G. (2017). Oropharyngeal Candidiasis: Fungal Invasion and Epithelial Cell Responses. PLoS Pathog..

[28] Finkel J.S., Xu W., Huang D., Hill E.M., Desai J.V., Woolford C.A., Nett J.E., Taff H., Norice C.T., Andes D.R. (2012). Portrait of *Candida albicans* adherence regulators. PLoS Pathog..

[29] Nobile C.J., Mitchell A.P. (2005). Regulation of cell-surface genes and biofilm formation by the *C. albicans* transcription factor Bcr1p. Curr. Biol..

[30] Sudbery P.E. (2011). Growth of *Candida albicans* hyphae. Nat. Rev. Microbiol..

[31] Konstantinidou N., Morrissey J.P. (2015). Co-occurence of filamentation defects and impaired biofilms in *Candida albicans* protein kinase mutants. FEMS Yeast Res..

[32] Rajendran R., May A., Sherry L., Kean R., Williams C., Jones B.L., Burgess K.V., Heringa J., Abeln S., Brandt B.W. (2016). Integrating *Candida albicans* metabolism with biofilm heterogeneity by transcriptome mapping. Sci. Rep..

[33] Al-Fattani M.A., Douglas L.J. (2006). Biofilm matrix of *Candida albicans* and *Candida tropicalis*: Chemical composition and role in drug resistance. J. Med. Microbiol..

[34] Nett J.E., Sanchez H., Cain M.T., Andes D.R. (2010). Genetic basis of *Candida* biofilm resistance due to drug-sequestering matrix glucan. J. Infect. Dis..

[35] Nobile C.J., Nett J.E., Hernday A.D., Homann O.R., Deneault J.-S., Nantel A., Andes D.R., Johnson A.D., Mitchell A.P. (2009). Biofilm matrix regulation by *Candida albicans* Zap1. PLoS Biol..

[36] Nett J.E., Sanchez H., Cain M.T., Ross K.M., Andes D.R. (2011). Interface of *Candida albicans* Biofilm Matrix-Associated Drug Resistance and Cell Wall Integrity Regulation. Eukaryot. Cell.

[37] Martins M., Uppuluri P., Thomas D.P., Cleary I.A., Henriques M., Lopez-Ribot J.L., Oliveira R. (2010). Presence of extracellular DNA in the *Candida albicans* biofilm matrix and its contribution to biofilms. Mycopathologia.

[38] Robbins N., Uppuluri P., Nett J., Rajendran R., Ramage G., Lopez-Ribot J.L., Andes D., Cowen L.E. (2011). Hsp90 Governs Dispersion and Drug Resistance of Fungal Biofilms. PLoS Pathog..

[39] Shapiro R.S., Uppuluri P., Zaas A.K., Collins C., Senn H., Perfect J.R., Heitman J., Cowen L.E. (2009). Hsp90 orchestrates temperature-dependent *Candida albicans* morphogenesis via Ras1-PKA signaling. Curr. Biol..

[40] Granger B.L. (2012). Insight into the anti-adhesive effect of yeast wall protein 1 of *Candida albicans*. Eukaryot. Cell.

[41] Banerjee M., Thompson D.S., Lazzell A., Carlisle P.L., Pierce C., Monteagudo C., Lopez-Ribot J.L., Kadosh D. (2008). *UME6*, a novel filament-specific regulator of *Candida albicans* hyphal extension and virulence. Mol. Biol. Cell.

[42] Braun B.R., Kadosh D., Johnson A.D. (2001). *NRG1*, a repressor of filamentous growth in *C. albicans*, is down-regulated during filament induction. EMBO J..

[43] Shen J., Cowen L.E., Griffin A.M., Chan L., Köhler J.R. (2008). The *Candida albicans* pescadillo homolog is required for normal hypha-to-yeast morphogenesis and yeast proliferation. Proc. Natl. Acad. Sci. USA.

[44] Pierce C.G., Vila T., Romo J.A., Montelongo-Jauregui D., Wall G., Ramasubramanian A., Lopez-Ribot J.L. (2017). The *Candida albicans* Biofilm matrix: Composition, structure and function. J. Fungi.

[45] Mukherjee P.K., Chandra J., Kuhn D.M., Ghannoum M.A. (2003). Mechanism of fluconazole resistance in *Candida albicans* biofilms: Phase-specific role of efflux pumps and membrane sterols. Infect. Immun..

[46] Hallstrom T.C., Lambert L., Schorling S., Balzi E., Goffeau A., Moye-Rowley W.S. (2001). Coordinate control of sphingolipid biosynthesis and multidrug resistance in Saccharomyces cerevisiae. J. Biol. Chem..

[47] Martin S.W., Konopka J.B. (2004). Lipid raft polarization contributes to hyphal growth in *Candida albicans*. Eukaryot. Cell.

[48] Toulmay A., Schneiter R. (2007). Lipid-dependent surface transport of the proton pumping ATPase: A model to study plasma membrane biogenesis in yeast. Biochimie.

[49] Lattif A.A., Chandra J., Chang J., Liu S., Zhou G., Chance M.R., Ghannoum M.A., Mukherjee P.K. (2008). Proteomics and pathway mapping analyses reveal phase-dependent over-expression of proteins associated with carbohydrate metabolic pathways in *Candida albicans* biofilms. Open Proteom. J..

[50] Yeater K.M., Chandra J., Cheng G., Mukherjee P.K., Zhao X., Rodriguez-Zas S.L., Kwast K.E., Ghannoum M.A., Hoyer L.L. (2007). Temporal analysis of *Candida albicans* gene expression during biofilm development. Microbiology.

[51] García R., Bermejo C., Grau C., Pérez R., Rodríguez-Peña J.M., Francois J., Nombela C., Arroyo J. (2004). The global transcriptional response to transient cell wall damage in Saccharomyces cerevisiae and its regulation by the cell integrity signaling pathway. J. Biol. Chem..

[52] Lattif A.A., Mukherjee P.K., Chandra J., Roth M.R., Welti R., Rouabhia M., Ghannoum M.A. (2011). Lipidomics of *Candida albicans* biofilms reveals phase-dependent production of phospholipid molecular classes and role for lipid rafts in biofilm formation. Microbiology.

[53] Hitchcock C.A., Barrett-Bee K.J., Russell N.J. (1987). The lipid composition and permeability to azole of an azole- and polyene-resistant mutant of *Candida albicans*. J. Med. Vet. Mycol..

[54] Liu G., Vellucci V.F., Kyc S., Hostetter M.K. (2009). Simvastatin inhibits *Candida albicans* biofilm in vitro. Pediatr. Res..

[55] Flemming H.C., Wingender J. (2010). The biofilm matrix. Nat. Rev. Microbiol..

[56] Baillie G.S., Douglas L.J. (2000). Matrix polymers of *Candida* biofilms and their possible role in biofilm resistance to antifungal agents. J. Antimicrob. Chemother..

[57] Zarnowski R., Sanchez H., Andes D.R. (2016). Large-scale production and isolation of *Candida* biofilm extracellular matrix. Nat. Protoc..

[58] Hornby J.M., Jensen E.C., Lisec A.D., Tasto J.J., Jahnke B., Shoemaker R., Dussault P., Nickerson K.W. (2001). Quorum sensing in the dimorphic fungus *Candida albicans* is mediated by farnesol. Appl. Environ. Microbiol..

[59] Singh A., Del Poeta M. (2011). Lipid signalling in pathogenic fungi. Cell. Microbiol..

[60] Calderone R.A. (2012). Candida and Candidiasis.

[61] Ramage G., Saville S.P., Wickes B.L., López-Ribot J.L. (2002). Inhibition of *Candida albicans* biofilm formation by farnesol, a quorum-sensing molecule. Appl. Environ. Microbiol..

[62] Katragkou A., McCarthy M., Alexander E.L., Antachopoulos C., Meletiadis J., Jabra-Rizk M.A., Petraitis V., Roilides E., Walsh T.J. (2014). In vitro interactions between farnesol and fluconazole, amphotericin B or micafungin against *Candida albicans* biofilms. J. Antimicrob. Chemother..

[63] Harris S.G., Padilla J., Koumas L., Ray D., Phipps R.P. (2002). Prostaglandins as modulators of immunity. Trends Immunol..

[64] Noverr M.C., Toews G.B., Huffnagle G.B. (2002). Production of prostaglandins and leukotrienes by pathogenic fungi. Infect. Immun..

[65] Alem M.A.S., Douglas L.J. (2005). Prostaglandin production during growth of *Candida albicans* biofilms. J. Med. Microbiol..

[66] Krause J., Geginat G., Tammer I. (2015). Prostaglandin E2 from *Candida albicans* stimulates the growth of Staphylococcus aureus in mixed biofilms. PLoS ONE.

[67] Alem M.A., Douglas L.J. (2004). Effects of aspirin and other nonsteroidal anti-inflammatory drugs on biofilms and planktonic cells of *Candida albicans*. Antimicrob. Agents Chemother..

[68] Harriott M.M., Noverr M.C. (2011). Importance of *Candida*–bacterial polymicrobial biofilms in disease. Trends Microbiol..

[69] Carlson E. (1982). Synergistic effect of *Candida albicans* and Staphylococcus aureus on mouse mortality. Infect. Immun..

[70] Adam B., Baillie G.S., Douglas L.J. (2002). Mixed species biofilms of *Candida albicans* and *Staphylococcus epidermidis*. J. Med. Microbiol..

[71] Taff H.T., Mitchell K.F., Edward J.A., Andes D.R. (2013). Mechanisms of Candida biofilm drug resistance. Future Microbiol..

[72] Law D., Moore C.B., Wardle H.M., Ganguli L.A., Keaney M.G., Denning D.W. (1994). High prevalence of antifungal resistance in *Candida* spp. from patients with AIDS. J. Antimicrob. Chemother..

[73] White T.C., Marr K.A., Bowden R.A. (1998). Clinical, cellular, and molecular factors that contribute to antifungal drug resistance. Clin. Microbiol. Rev..

[74] White T.C. (1997). Increased mRNA levels of ERG16, CDR, and MDR1 correlate with increases in azole resistance in *Candida albicans* isolates from a patient infected with human immunodeficiency virus. Antimicrob. Agents Chemother..

[75] Marichal P., Koymans L., Willemsens S., Bellens D., Verhasselt P., Luyten W., Borgers M., Ramaekers F.C., Odds F.C., Bossche H.V. (1999). Contribution of mutations in the cytochrome P450 14α-demethylase (Erg11p, Cyp51p) to azole resistance in *Candida albicans*. Microbiology.

[76] Sanglard D., Kuchler K., Ischer F., Pagani J.L., Monod M., Bille J. (1995). Mechanisms of resistance to azole antifungal agents in *Candida albicans* isolates from AIDS patients involve specific multidrug transporters. Antimicrob. Agents Chemother..

[77] Albertson G.D., Niimi M., Cannon R.D., Jenkinson H.F. (1996). Multiple efflux mechanisms are involved in *Candida albicans* fluconazole resistance. Antimicrob. Agents Chemother..

[78] Sanglard D., Ischer F., Koymans L., Bille J. (1998). Amino acid substitutions in the cytochrome P-450 lanosterol 14α-demethylase (CYP51A1) from azole-resistant *Candida albicans* clinical isolates contribute to resistance to azole antifungal agents. Antimicrob. Agents Chemother..

[79] Sanglard D., Ischer F., Monod M., Bille J. (1996). Susceptibilities of *Candida albicans* multidrug transporter mutants to various antifungal agents and other metabolic inhibitors. Antimicrob. Agents Chemother..

[80] Lamb D.C., Kelly D.E., Schunck W.-H., Shyadehi A.Z., Akhtar M., Lowe D.J., Baldwin B.C., Kelly S.L. (1997). The mutation T315A in *Candida albicans* sterol 14α-demethylase causes reduced enzyme activity and fluconazole resistance through reduced affinity. J. Biol. Chem..

[81] Kelly S.L., Lamb D.C., Corran A.J., Baldwin B.C., Kelly D.E. (1995). Mode of Action and Resistance to Azole Antifungals Associated with the Formation of 14α-Methylergosta-8,24(28)-dien-3β,6α-diol. Biochem. Biophys. Res. Commun..

[82] Lupetti A., Danesi R., Campa M., Tacca M.D., Kelly S. (2002). Molecular basis of resistance to azole antifungals. Trends Mol. Med..

[83] Hitchcock C.A., Barrett-Bee K.J., Russell N.J. (1989). The lipid composition and permeability to the triazole antifungal antibiotic ICI 153066 of serum-grown mycelial cultures of *Candida albicans*. J. Gen. Microbiol..

[84] García-Sánchez S., Aubert S., Iraqui I., Janbon G., Ghigo J.-M., d’Enfert C. (2004). *Candida albicans* biofilms: A developmental state associated with specific and stable gene expression patterns. Eukaryot. Cell.

[85] Nett J.E., Lepak A.J., Marchillo K., Andes D.R. (2009). Time course global gene expression analysis of an in vivo *Candida* biofilm. J. Infect. Dis..

[86] Elias S., Banin E. (2012). Multi-species biofilms: Living with friendly neighbors. FEMS Microbiol. Rev..

[87] Pulimood S., Ganesan L., Alangaden G., Chandrasekar P. (2002). Polymicrobial candidemia. Diagn. Microbiol. Infect. Dis..

[88] Klotz S.A., Chasin B.S., Powell B., Gaur N.K., Lipke P.N. (2007). Polymicrobial bloodstream infections involving *Candida* species: Analysis of patients and review of the literature. Diagn. Microbiol. Infect. Dis..

[89] Kim S.-H., Yoon Y.K., Kim M.J., Sohn J.W. (2013). Risk factors for and clinical implications of mixed *Candida*/bacterial bloodstream infections. Clin. Microbiol. Infect..

[90] Jack A.A., Daniels D.E., Jepson M.A., Vickerman M.M., Lamont R.J., Jenkinson H.F., Nobbs A.H. (2015). *Streptococcus gordonii* comCDE (competence) operon modulates biofilm formation with *Candida albicans*. Microbiology.

[91] Jarosz L.M., Deng D.M., van der Mei H.C., Crielaard W., Krom B.P. (2009). *Streptococcus mutans* competence-stimulating peptide inhibits *Candida albicans* hypha formation. Eukaryot. Cell.

[92] de Repentigny J., Lévesque R., Mathieu L.G. (1979). Increase in the in vitro susceptibility of *Staphylococcus aureus* to antimicrobial agents in the presence of *Candida albicans*. Can. J. Microbiol..

[93] Harriott M.M., Noverr M.C. (2009). *Candida albicans* and *Staphylococcus aureus* Form Polymicrobial Biofilms: Effects on Antimicrobial Resistance. Antimicrob. Agents Chemother..

[94] Budtz-Jørgensen E., Mojon P., Banon-Clément J.M., Baehni P. (1996). Oral candidosis in long-term hospital care: Comparison of edentulous and dentate subjects. Oral Dis..

[95] AL-Dwairi Z.N. (2008). Prevalence and risk factors associated with denture-related stomatitis in healthy subjects attending a dental teaching hospital in North Jordan. J. Ir. Dent. Assoc..

[96] Verran J., Motteram K.L. (1987). The effect of adherent oral streptococci on the subsequent adherence of *Candida albicans* to acrylic in vitro. J. Dent..

[97] Branting C., Sund M.L., Linder L.E. (1989). The influence of *Streptococcus mutans* on adhesion of *Candida albicans* to acrylic surfaces in vitro. Arch. Oral Biol..

[98] Inoue Y., Shiraishi A., Hada T., Hirose K., Hamashima H., Shimada J. (2004). The antibacterial effects of terpene alcohols on *Staphylococcus aureus* and their mode of action. FEMS Microbiol. Lett..

[99] Kim D., Sengupta A., Niepa T.H.R., Lee B.-H., Weljie A., Freitas-Blanco V.S., Murata R.M., Stebe K.J., Lee D., Koo H. (2017). *Candida albicans* stimulates *Streptococcus mutans* microcolony development via cross-kingdom biofilm-derived metabolites. Sci. Rep..

[100] Fernandes R.A., Monteiro D.R., Arias L.S., Fernandes G.L., Delbem A.C.B., Barbosa D.B. (2016). Biofilm formation by *Candida albicans* and *Streptococcus mutans* in the presence of farnesol: A quantitative evaluation. Biofouling.

[101] Rittershaus P.C., Kechichian T.B., Allegood J.C., Merrill A.H., Hennig M., Luberto C., Del Poeta M. (2006). Glucosylceramide synthase is an essential regulator of pathogenicity of *Cryptococcus neoformans*. J. Clin. Investig..

[102] Dickson R.C., Naglee E.E., Wells G.B., Naglee M.M., Lester R.L. (1997). Synthesis of mannose-(inositol-P)2-ceramide, the major sphingolipid in *Saccharomyces cerevisiae*, requires the *IPT1* (YDR072c) gene. J. Biol. Chem..

[103] Alfatah M., Bari V.K., Nahar A.S., Bijlani S., Ganesan K. (2017). Critical role for *CaFEN1* and *CaFEN12* of *Candida albicans* in cell wall integrity and biofilm formation. Sci. Rep..

[104] Cassilly C.D., Farmer A.T., Montedonico A.E., Smith T.K., Campagna S.R., Reynolds T.B. (2017). Role of phosphatidylserine synthase in shaping the phospholipidome of *Candida albicans*. FEMS Yeast Res..

[105] Chen Y.-L., Montedonico A.E., Kauffman S., Dunlap J.R., Menn F.-M., Reynolds T.B. (2010). Phosphatidylserine synthase and phosphatidylserine decarboxylase are essential for cell wall integrity and virulence in *Candida albicans*. Mol. Microbiol..

[106] Mukherjee P.K., Sheehan D.J., Hitchcock C.A., Ghannoum M.A. (2005). Combination treatment of invasive fungal infections. Clin. Microbiol. Rev..

[107] Bruzual I., Riggle P., Hadley S., Kumamoto C.A. (2007). Biofilm formation by fluconazole-resistant *Candida albicans* strains is inhibited by fluconazole. J. Antimicrob. Chemother..

[108] Bink A., Kucharíková S., Neirinck B., Vleugels J., Van Dijck P., Cammue B.P.A., Thevissen K. (2012). The Nonsteroidal Antiinflammatory Drug Diclofenac Potentiates the In Vivo Activity of Caspofungin Against *Candida albicans* Biofilms. J. Infect. Dis..

[109] Khan M.S.A., Ahmad I. (2012). Antibiofilm activity of certain phytocompounds and their synergy with fluconazole against *Candida albicans* biofilms. J. Antimicrob. Chemother..

[110] Seigneuret M., Devaux P.F. (1984). ATP-dependent asymmetric distribution of spin-labeled phospholipids in the erythrocyte membrane: Relation to shape changes. Proc. Natl. Acad. Sci. USA.

[111] Daleke D.L., Huestis W.H. (1989). Erythrocyte morphology reflects the transbilayer distribution of incorporated phospholipids. J. Cell Biol..

[112] Devaux P.F. (1991). Static and dynamic lipid asymmetry in cell membranes. Biochemistry.

[113] Zachowski A. (1993). Phospholipids in animal eukaryotic membranes: Transverse asymmetry and movement. Biochem. J..

[114] Murate M., Abe M., Kasahara K., Iwabuchi K., Umeda M., Kobayashi T. (2015). Transbilayer distribution of lipids at nano scale. J. Cell Sci..

[115] Yabas M., Coupland L.A., Cromer D., Winterberg M., Teoh N.C., D’Rozario J., Kirk K., Bröer S., Parish C.R., Enders A. (2014). Mice deficient in the putative phospholipid flippase ATP11C exhibit altered erythrocyte shape, anemia, and reduced erythrocyte life span. J. Biol. Chem..

[116] Naito T., Takatsu H., Miyano R., Takada N., Nakayama K., Shin H.-W. (2015). Phospholipid flippase ATP10A translocates phosphatidylcholine and is involved in plasma membrane dynamics. J. Biol. Chem..

[117] Takada N., Naito T., Inoue T., Nakayama K., Takatsu H., Shin H. (2018). Phospholipid-flipping activity of P4-ATPase drives membrane curvature. EMBO J..

[118] Srivastava A., Sircaik S., Husain F., Thomas E., Ror S., Rastogi S., Alim D., Bapat P., Andes D.R., Nobile C.J. (2017). Distinct roles of the 7-transmembrane receptor protein Rta3 in regulating the asymmetric distribution of phosphatidylcholine across the plasma membrane and biofilm formation in *Candida albicans*. Cell. Microbiol..

